# *Carollia perspicillata*: A Small Bat with Tremendous Translational Potential for Studies of Brain Aging and Neurodegeneration

**DOI:** 10.3390/biomedicines9101454

**Published:** 2021-10-13

**Authors:** Mark Stewart, Timothy Morello, Richard Kollmar, Rena Orman

**Affiliations:** 1 Departments of Physiology & Pharmacology, SUNY Downstate Health Sciences University, Brooklyn, NY 11203, USA; mark.stewart@downstate.edu (M.S.); timothy.morello@downstate.edu (T.M.); 2 Departments of Neurology, SUNY Downstate Health Sciences University, Brooklyn, NY 11203, USA; 3 Departments of Cell Biology, SUNY Downstate Health Sciences University, Brooklyn, NY 11203, USA; richard.kollmar@downstate.edu; 4 Departments of Otolaryngology, SUNY Downstate Health Sciences University, Brooklyn, NY 11203, USA

**Keywords:** prosubiculum, calbindin, calretinin, parvalbumin, fruit bat

## Abstract

As the average human lifespan lengthens, the impact of neurodegenerative disease increases, both on the individual suffering neurodegeneration and on the community that supports those individuals. Studies aimed at understanding the mechanisms of neurodegeneration have relied heavily on observational studies of humans and experimental studies in animals, such as mice, in which aspects of brain structure and function can be manipulated to target mechanistic steps. An animal model whose brain is structurally closer to the human brain, that lives much longer than rodents, and whose husbandry is practical may be valuable for mechanistic studies that cannot readily be conducted in rodents. To demonstrate that the long-lived Seba’s short-tailed fruit bat, *Carollia perspicillata*, may fit this role, we used immunohistochemical labeling for NeuN and three calcium-binding proteins, calretinin, parvalbumin, and calbindin, to define hippocampal formation anatomy. Our findings demonstrate patterns of principal neuron organization that resemble primate and human hippocampal formation and patterns of calcium-binding protein distribution that help to define subregional boundaries. Importantly, we present evidence for a clear prosubiculum in the bat brain that resembles primate prosubiculum. Based on the similarities between bat and human hippocampal formation anatomy, we suggest that *Carollia* has unique advantages for the study of brain aging and neurodegeneration. A captive colony of *Carollia* allows age tracking, diet and environment control, pharmacological manipulation, and access to behavioral, physiological, anatomical, and molecular evaluation.

## 1. Introduction

As the world’s population ages, the public health impact of neurodegenerative disease increases. Understanding the reasons for age-related neurodegeneration and discovering mechanisms by which neurons are protected are important in addressing this growing crisis. Past studies of brain aging can be broadly grouped into two categories: (1) observational studies of anatomy, physiology, molecular biology, and behavior at different ages or stages of disease in a large variety of species, and (2) experimental studies, predominantly in mice, that evaluate the same variables but with direct manipulation of genes, proteins or pharmacology to simulate, accelerate or reverse neurodegeneration. Whereas this work has led to considerable progress [1,2], the numerous differences between mouse brain and human brain have limited the translation of experimental findings in mice for diagnostic or therapeutic tools for humans [3,4]. 

The hippocampal formation in humans and some rodent models is severely impacted by neurodegenerative disease (e.g., [5,6,7,8,9,10,11,12]). Age-related neuronal loss has been shown to impact inhibitory neuronal in multiple brain regions [13,14,15,16,17]. The calcium-binding proteins, parvalbumin, calbindin, and calretinin, which label inhibitory neurons in many brain structures [18,19,20], are valuable markers for age-related changes in inhibitory circuits.

There have been studies of aspects of hippocampal formation anatomy and physiology in bats, including animals from *Myotis*, *Rousettus*, and *Epomophorus* (e.g., [21,22,23]), but the hippocampal formation of *Carollia* has not been described in detail [24,25]. Here we present anatomical details of *Carollia* hippocampal formation using immunohistochemistry for NeuN, calretinin, parvalbumin, and calbindin, including evidence of a clear prosubiculum, a subregion that is otherwise best seen in the primate brain [26,27,28,29]. 

We propose the bat as a novel model for studies of the neurobiology of aging and neurodegenerative disease. Bats demonstrate extreme longevity [30,31,32,33,34], making them an excellent animal model of aging. The short-tailed fruit bat, *Carollia perspicillata*, lives up to 13 years in captivity [35,36,37], remains reproductively active throughout life (females) [38,39], and its neuroanatomy resembles that of primates more closely than the neuroanatomy of rodents does (e.g., [24,25,28,40,41,42,43]). Importantly, *Carollia* are small in body size and their husbandry has been refined sufficiently that they are practical to maintain in captivity [35,36,37]. 

## 2. Methods

ANIMALS AND HUSBANDRY: As described previously [24,40,44], our use of animals conformed to the standards set forth in the NIH Guide for the Care and Use of Laboratory Animals [45] and was approved by the SUNY Downstate Institutional Animal Care and Use Committee. 

Our colony, which has been in existence >30 years, consists of approximately 150 members with active breeding that can be controlled and timed. Our basic husbandry of *Carollia* consists of a caging system with open feeding and closed roosting sections of 20 ft^3^ and 16.7 ft^3^, respectively [35,36,37]. We keep animals in three configurations: male animals only, female animals only, and mixed gender. Ten to twenty animals are housed in each cage with continuous access to water. The room is kept on a 12:12 light:dark cycle. Animals receive a blended fruit mix that contains apricot nectar, peaches, monkey chow, supplemental vitamins and minerals, and emulsified corn oil [46]. The diet is periodically enriched with pieces of whole fruits. The daily per diem housing cost for bats at our institution is identical to the cost for mice. The food costs are somewhat higher because of the cost differential between our blended fruit mix ingredients and standard rodent chow, but the costs of our food resemble the cost of specialized rodent diets (approximately $50–60/animal/year). 

EXPERIMENTAL DESIGN: We collected 4 sagittal and 2 coronal series of brain sections using NeuN as a general neuronal marker from 6 adult bats (4 female and 2 male). Animals ranged in age from 4–12 months of age and had a body mass range of 15–25 g. On serially adjacent or near-adjacent (within 75 µm) sections in 2 brains, NeuN labeling was paired with labeling for 1 of 3 calcium-binding proteins—calretinin, parvalbumin, or calbindin. 

IMMUNOHISTOCHEMISTRY: Each animal was anesthetized with urethane (0.02–0.04 mL 20% wt/vol solution in water per animal given subcutaneously) and perfused with cold phosphate-buffered saline (PBS, 0.1 M, pH 7.4: NaCl 1.37 *×* 10^−1^ M, KCl 2.68 × 10^−3^ M, Na_2_HPO_4_ 1.014 × 10^−2^ M, KH_2_PO_4_ 1.76 × 10^−3^ M), followed by cold 4% wt/vol paraformaldehyde in PBS. After a variable post-fixation period depending upon the planned staining, brains were washed multiple times and cryoprotected with 30% sucrose. Sagittal or coronal sections were cut at 35 µm thickness with a freezing microtome (Thermo Fisher Scientific Microm HM 430, Waltham, MA, USA). Sections were collected into wells containing PBS (pH 7.2, NaCl 1.54 × 10−1 M, Na2HPO4 7.68 × 10−3 M, NaH2PO4 9.08 × 10−3 M), and stored at 4 °C until processed. Sections were incubated with primary antibodies overnight (16–20 h) at 4 °C. The primary antibodies included mono- and polyclonal antibodies against NeuN, parvalbumin, calbindin, and calretinin (see Table 1 of antibodies for sources and RRID numbers of primary and secondary antibodies). After washing with PBS, secondary antibodies, diluted in blocking buffer, were added and incubated in the dark for 2 h. After washing, sections were counterstained with 4′,6-diamidino-2-phenylindole (DAPI) (Invitrogen, D357) for 20 min and mounted with ProLong Diamond Antifade Mountant (Invitrogen, P36961).

There was no labeling in the absence of primary antibody. Our controls for primary specificity include (a) matches between the sequenced bat genomes [47] and the sequences of antigens used for antibody production (published by supplier), (b) detection of the correct molecular weight band on Western blots of brain tissue, and (c) similar labeling patterns in bats as have been published in other species.

IMAGING: Samples were imaged with an Axio Observer 7/LSM 800 inverted compound microscope and Zen Blue version 2.3 software (Carl Zeiss Microscopy, Thornwood, NY, USA). Sections were imaged with 5×/0.16 and 10×/0.45 plan-apochromatic objectives in widefield mode. Details were imaged with a 63×/1.4 plan-apochromatic objective in confocal mode. Tiled 63× overview images were taken with tile sizes at 512 × 512, pixel dwell at 1 µs, 2 times line averaging, and the pinhole set to 1 Airy unit. Laser intensity and master gain were adjusted for optimal contrast per antibody. 

## 3. Results

The brain of *Carollia* resembles the mouse brain in its overall size. A large cerebellum is located behind the forebrain [25], and the rostro-caudal extent of the adult forebrain is about 10 mm and its largest dimension from side to side is about 9 mm. 

Previously, we published serial images of *Carollia* brains that were sectioned in coronal [25] and sagittal [24] planes, and gave basic identification of hippocampal formation regions in each instance. An important feature that can be extracted from these series is that the main axis of the dorsal hippocampal formation in *Carollia* forms an angle that is approximately 30 degrees off the coronal plane (running from rostro-medial to caudo-lateral) and approximately 60 degrees off the sagittal plane. Neither plane of section, therefore, cuts an ideal transverse section, but the sagittal plane is closer to a transverse plane of section for dorsal hippocampal formation in *Carollia* brain. 

Two other advantages of the sagittal plane of section warrant comment. The first is that the greater roundness of the *Carollia* brain [24,25] compared with rodent brain [41,42] can make replicating coronal, horizontal, or other oblique (e.g., perfectly transverse for the main axis of the dorsal hippocampal formation) planes of section challenging, whereas there is never a question about reproducing the sagittal plane of section. Second, as described below, the modest tilt of the sagittal plane of section off what would be an ideal transverse plane results in a favorable elongation of the hippocampal cross-section that facilitates subregional definition.

**Figure 1:**
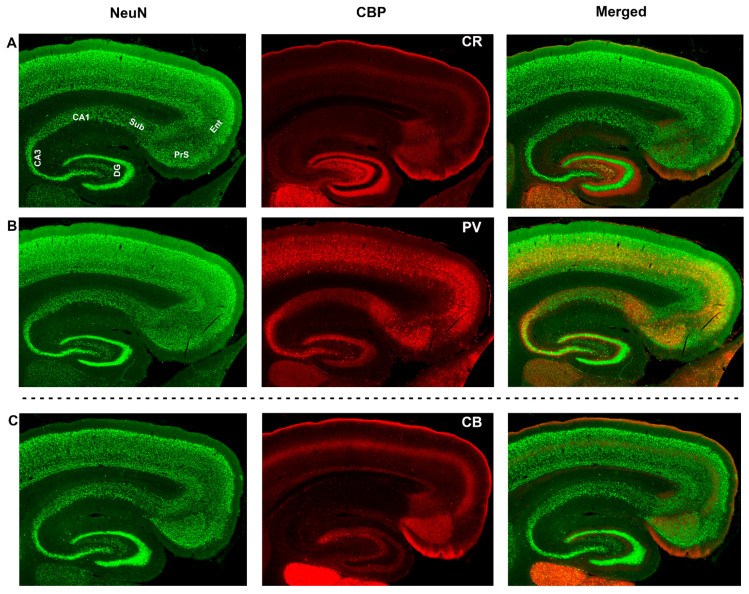
Hippocampal formation in the brain of *Carollia perspicillata* labeled with NeuN, calretinin, parvalbumin, and calbindin. Widefield images of sagittal sections of Carollia brain showing three pairs of labeling (by row) with NeuN and a calcium binding protein (CBP). Top row (**A**): NeuN with calretinin. Subregional labels are indicated on the NeuN only section (top left). Calretinin labeling alone is shown in the top center (CBP/CR) and the merged image is shown at the top right. Middle row (**B**): NeuN and parvalbumin (PV) labeling from the adjacent section. Bottom row (**C**): NeuN and calbindin (CB) labeling from a near-adjacent section from the same brain. Dotted-line separator indicates that the bottom row is not adjacent to the middle row. The distributions of the calcium binding proteins help to delineate regions and to highlight differences in the distributions of inhibitory neurons and processes. Additional abbreviations: DG—dentate gyrus, CA3—part of regio inferior of cornu Ammonis, CA1—regio superior of cornu Ammonis, Sub—subiculum, PrS—presubiculum, Ent—entorhinal cortex.

Figure 1 illustrates NeuN labeling of sections of dorsal hippocampal formation. In each, NeuN was paired with one of three calcium binding proteins—calretinin, parvalbumin, or calbindin. The basic hippocampal subregional identifications can be made and are illustrated using the changes in density of NeuN labeled neurons, layer thickness, and the change in appearance from 3-layered (dentate gyrus, CA3, CA1, subiculum) to 6-layered cortex (presubiculum, entorhinal cortex, neocortex). The labeling patterns for the three calcium binding proteins demonstrate the different distributions of labeled cells and processes in each subregion, but also reinforce subregional boundary identification. 

Calretinin and closely related calbindin clearly label dentate gyrus, hilus, and help to differentiate the upper and lower blades of dentate (especially calbindin). Calretinin and calbindin also strongly label the presubiculum and entorhinal cortex. Both markers label cortical layer I, and calbindin labeling is more pronounced in the deeper layers, which helps to distinguish parasubiculum in between presubiculum and entorhinal cortex.

**Figure 2:**
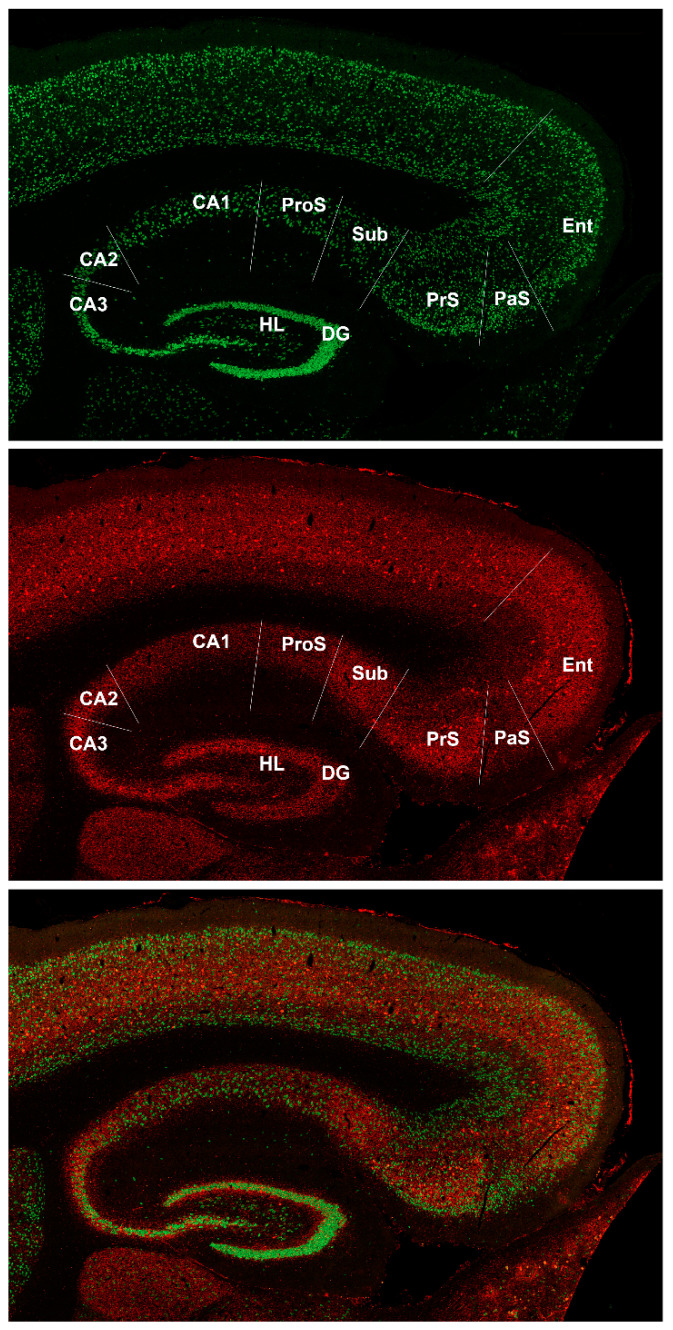
Hippocampal formation in the brain of *Carollia perspicillata* labeled with NeuN and parvalbumin. Tiled confocal 63× sagittal composite section of Carollia brain labeled with NeuN (top panel—green channel) and parvalbumin (middle panel—red channel) to show subregional boundaries in greater detail. A merged image is shown in the bottom panel. The subregional boundaries are described in the Results and are drawn on the figure as white lines. The sagittal plane of this section was determined to be 2.45 mm from the lateral edge and 2.07 mm from the midline. Additional abbreviations: DG—dentate gyrus, HL—dentate hilus, CA3—part of regio inferior of cornu Ammonis, CA2—part of regio inferior of cornu Ammonis, CA1—regio superior of cornu Ammonis, ProS—prosubiculum, Sub—subiculum, PrS—presubiculum, PaS—parasubiculum, Ent—entorhinal cortex.

Parvalbumin is arguably the most valuable label in defining dorsal hippocampal formation subregional boundaries in *Carollia* brain (Figure 2). The density of parvalbumin-immunoreactive fibers is evidently heavier in CA3 compared with CA1 with pyramidal cells (appearing as “holes” in the parvalbumin image) enwrapped by labeled processes. CA2 can be distinguished from its neighbor, CA3, on the basis of increased spread of parvalbumin-labeled processes into the apical dendritic zone, and the change in packing of NeuN-labeled cells. CA2 can be distinguished from CA1 on the basis of the much lower density of parvalbumin-labeled processes and the distinctly looser packing of CA1 neurons. 

Coming from the entorhinal cortex in toward Ammon’s horn, parvalbumin labeling identifies the parasubiculum as a wedge of cortex in between entorhinal cortex and presubiculum with relatively lower level of parvalbumin labeling that distinguishes parasubiculum. The heavily labeled presubiculum abruptly transitions into the broad 3-layer cortex of subiculum (Figure 2).

Parvalbumin also reveals a prosubiculum in *Carollia* brain. The prosubiculum has a cell-layer breadth and packing density that resembles the subiculum (adjacent to the presubiculum) but contrasts with CA1. The differentiation of prosubiculum from subiculum can be made by parvalbumin labeling: the subiculum is much more heavily labeled than prosubiculum, and the separation of presubiculum from subiculum is obvious in the figure.

## 4. Discussion

The brain of *Carollia perspicillata* is remarkable for many reasons. Here, we illustrate the basic anatomy of the dorsal hippocampal formation. Key features are (1) the compact cell layer (along the alvear–pial axis) in area CA3 in contrast to the broader cell layer of area CA1 and (2) the presence of a clear prosubiculum, both of which are prominent features of primate brains, but not rodent brains. These features strongly support the argument that *Carollia* brain is closer in neuroanatomical features and organization to human brain than rodent brain is to human brain (see also [43,48]). The relative size, the dorso-ventral extent, and the location of the hippocampal formation in relation to the dorsal surface of the brain give *Carollia* hippocampal formation the experimental access advantages of rodent hippocampus. The long natural life span of *Carollia* (approximately 13 years in captivity) and the ability to maintain these animals in controlled conditions lead us to conclude that *Carollia* is a unique model for studies of many neurological issues including brain aging and neurodegeneration.

### 4.1. Prosubiculum in Bat Brain

Prosubiculum is not labeled in the majority of reports on rodent hippocampal formation structure, including our own (e.g., [41,42,49,50,51,52,53,54]). As such, specific functional attributes of prosubicular cells and the network connectivity of prosubiculum are not well understood. Prosubiculum has, however, been distinguished from subiculum in rodent brain using transcriptomics [55] and in situ hybridization data [56], and mouse and human may be more similar than they first appear [28,56].

Our data show a very clear separation of prosubiculum from both CA1 and subiculum and that prosubiculum in sagittal sections of *Carollia* brain is of sufficient size for studies of cell function and connectivity. Our boundaries compare to those reported with a host of other markers in rodent and primate brain [28]. Clear subregional identification is invaluable because subiculum is among the earliest structures to be impacted in Alzheimer’s disease [7] and prosubiculum has been identified as a specific target for amyloid plaque deposition [29].

Due to the dorso-ventral orientation of the hippocampus in the bat, as opposed to the more rostro-caudal orientation of the hippocampus in the rodent, there is no plane of section in rat or mouse that can be used as a simple comparison to the bat. However, there are numerous examples in atlases, publications, and websites with sections from mouse and rat brain if one was interested in attempting such comparisons for overall structure or more specific comparisons of specific labels.

### 4.2. Carollia as a New Animal Model of Brain Aging and Neurodegeneration

The neuroanatomical features of *Carollia* hippocampal formation and its natural long life makes *Carollia* an important alternative model for studies of the neurobiology of aging, neurodegenerative disease, and normal hippocampal function. 

It is critically important to answer the question: are bats subject to neurodegenerative diseases? Given that bats naturally live much longer (>10 years) than rats and mice that are commonly used as research models (typically up to 3 years old), there is much more time for cumulative evidence of neurodegenerative disease. This might make bats a valuable model for neuropathology. If, on the other hand, evidence of neurodegenerative disease in such aged animals is absent, bats’ resistance to neurodegenerative changes will add to their appeal as a model for longevity.

A critical feature of *Carollia* in favor of its utility as an animal model is that these animals can be easily kept in captivity, which offers a controlled environment with the ability to control stress levels, diet and other community variables that can influence normal aging and the development of neurodegenerative disease. Whether bats represent a better animal model of neurodegenerative disease or the resistance to neurodegenerative disease is a focus of our work, and the answer will help to define the utility of the bat. 

## Figures and Tables

**Table 1:** Table of Antibodies.

Target	Species	Immunogen	Clonality	Isotype	Working Dilution	Source (Cat. #)	RRID
PRIMARY ANTIBODIES							
Calbindin	Rabbit	recombinant rat calbindin D-28k	Poly	(antiserum)	1/3000	Swant (CB 38)	AB_10000340
Calretinin	Rabbit	recombinant human calretinin containing a 6-his tag at the N-terminal	Poly	(antiserum)	1/3000	Swant (CR7697)	AB_2619710
NeuN	Mouse	purified cell nuclei from mouse brain	Mono	IgG1	1/2000	Millipore (MAB377)	AB_2298772
Parvalbumin	Rabbit	recombinant rat parvalbumin	Poly	(antiserum)	1/3000	Swant (PV 27)	AB_2631173
SECONDARY ANTIBODIES							
Mouse IgG	Goat		Poly	IgG	1/500	Jackson (115-545-003)	AB_2338840
Rabbit IgG	Goat		Poly	IgG	1/500	Jackson (111-295-003)	AB_2338022

## Data Availability

Data images available upon appropriate request.

## References

[1] Beach T.G. (2017). A Review of Biomarkers for Neurodegenerative Disease: Will They Swing Us Across the Valley?. Neurol. Ther..

[2] Ramachandran A.K., Das S., Joseph A., Shenoy G.G., Alex A.T., Mudgal J. (2021). Neurodegenerative Pathways in Alzheimer’s Disease: A Review. Curr. Neuropharmacol..

[3] Driscoll I., Sutherland R.J. (2005). The aging hippocampus: Navigating between rat and human experiments. Rev. Neurosci..

[4] Stewart M. (2021). And when I die… What time should I expect it?. J. Physiol..

[5] Trujillo-Estrada L., Davila J.C., Sanchez-Mejias E., Sanchez-Varo R., Gomez-Arboledas A., Vizuete M., Vitorica J., Gutierrez A. (2014). Early neuronal loss and axonal/presynaptic damage is associated with accelerated amyloid-beta accumulation in AbetaPP/PS1 Alzheimer’s disease mice subiculum. J. Alzheimers’ Dis..

[6] Angulo S.L., Orman R., Neymotin S.A., Liu L., Buitrago L., Cepeda-Prado E., Stefanov D., Lytton W.W., Stewart M., Small S.A. (2017). Tau and amyloid-related pathologies in the entorhinal cortex have divergent effects in the hippocampal circuit. Neurobiol. Dis..

[7] Carlesimo G.A., Piras F., Orfei M.D., Iorio M., Caltagirone C., Spalletta G. (2015). Atrophy of presubiculum and subiculum is the earliest hippocampal anatomical marker of Alzheimer’s disease. Alzheimers’ Dement..

[8] Ma C., Wang G.Z., Braak H. (1994). Pathological changes of the retrosplenial cortex in senile dementia of Alzheimer type. Chin. Med. J..

[9] Pengas G., Williams G.B., Acosta-Cabronero J., Ash T.W., Hong Y.T., Izquierdo-Garcia D., Fryer T.D., Hodges J.R., Nestor P.J. (2012). The relationship of topographical memory performance to regional neurodegeneration in Alzheimer’s disease. Front. Aging Neurosci..

[10] Nestor P.J., Fryer T.D., Ikeda M., Hodges J.R. (2003). Retrosplenial cortex (BA 29/30) hypometabolism in mild cognitive impairment (prodromal Alzheimer’s disease). Eur. J. Neurosci..

[11] Robertson R.T., Baratta J., Yu J., LaFerla F.M. (2009). Amyloid-beta expression in retrosplenial cortex of triple transgenic mice: Relationship to cholinergic axonal afferents from medial septum. Neuroscience.

[12] Fisher E.M.C., Bannerman D.M. (2019). Mouse models of neurodegeneration: Know your question, know your mouse. Sci. Transl. Med..

[13] Fuhrer T.E., Palpagama T.H., Waldvogel H.J., Synek B.J.L., Turner C., Faull R.L., Kwakowsky A. (2017). Impaired expression of GABA transporters in the human Alzheimer’s disease hippocampus, subiculum, entorhinal cortex and superior temporal gyrus. Neuroscience.

[14] Kwakowsky A., Calvo-Flores Guzman B., Pandya M., Turner C., Waldvogel H.J., Faull R.L. (2018). GABAA receptor subunit expression changes in the human Alzheimer’s disease hippocampus, subiculum, entorhinal cortex and superior temporal gyrus. J. Neurochem..

[15] Mikkonen M., Alafuzoff I., Tapiola T., Soininen H., Miettinen R. (1999). Subfield- and layer-specific changes in parvalbumin, calretinin and calbindin-D28K immunoreactivity in the entorhinal cortex in Alzheimer’s disease. Neuroscience.

[16] Ahn J.H., Hong S., Park J.H., Kim I.H., Cho J.H., Lee T.K., Lee J.C., Chen B.H., Shin B.N., Bae E.J. (2017). Immunoreactivities of calbindinD28k, calretinin and parvalbumin in the somatosensory cortex of rodents during normal aging. Mol. Med. Rep..

[17] Freund T.F., Buzsaki G. (1996). Interneurons of the hippocampus. Hippocampus.

[18] Petilla Interneuron Nomenclature G., Ascoli G.A., Alonso-Nanclares L., Anderson S.A., Barrionuevo G., Benavides-Piccione R., Burkhalter A., Buzsaki G., Cauli B., Defelipe J. (2008). Petilla terminology: Nomenclature of features of GABAergic interneurons of the cerebral cortex. Nat. Rev. Neurosci..

[19] DeFelipe J., Lopez-Cruz P.L., Benavides-Piccione R., Bielza C., Larranaga P., Anderson S., Burkhalter A., Cauli B., Fairen A., Feldmeyer D. (2013). New insights into the classification and nomenclature of cortical GABAergic interneurons. Nat. Rev. Neurosci..

[20] Baimbridge K.G., Celio M.R., Rogers J.H. (1992). Calcium-binding proteins in the nervous system. Trends Neurosci..

[21] Eliav T., Geva-Sagiv M., Yartsev M.M., Finkelstein A., Rubin A., Las L., Ulanovsky N. (2018). Nonoscillatory Phase Coding and Synchronization in the Bat Hippocampal Formation. Cell.

[22] Gatome C.W., Mwangi D.K., Lipp H.P., Amrein I. (2010). Hippocampal neurogenesis and cortical cellular plasticity in Wahlberg’s epauletted fruit bat: A qualitative and quantitative study. Brain Behav. Evol..

[23] Cotter J.R., Laemle L.K. (1990). Cholecystokinin (CCK)-like immunoreactivity in the brain of the little brown bat (*Myotis lucifugus*). J. Hirnforsch.

[24] Orman R., Kollmar R., Stewart M. (2017). Claustrum of the short-tailed fruit bat, *Carollia perspicillata*: Alignment of cellular orientation and functional connectivity. J. Comp. Neurol..

[25] Scalia F., Rasweiler J.J., Scalia J., Orman R., Stewart M. (2013). Forebrain Atlas of the Short-Tailed Fruit Bat, Carollia perpicillata.

[26] Blatt G.J., Rosene D.L. (1998). Organization of direct hippocampal efferent projections to the cerebral cortex of the rhesus monkey: Projections from CA1, prosubiculum, and subiculum to the temporal lobe. J. Comp. Neurol..

[27] Braak H., Del Tredici K. (2020). From the Entorhinal Region via the Prosubiculum to the Dentate Fascia: Alzheimer Disease-Related Neurofibrillary Changes in the Temporal Allocortex. J. Neuropathol. Exp. Neurol..

[28] Ding S.L. (2013). Comparative anatomy of the prosubiculum, subiculum, presubiculum, postsubiculum, and parasubiculum in human, monkey, and rodent. J. Comp. Neurol..

[29] Marshall G.A., Kaufer D.I., Lopez O.L., Rao G.R., Hamilton R.L., DeKosky S.T. (2004). Right prosubiculum amyloid plaque density correlates with anosognosia in Alzheimer’s disease. J. Neurol. Neurosurg. Psychiatry.

[30] Podlutsky A.J., Khritankov A.M., Ovodov N.D., Austad S.N. (2005). A new field record for bat longevity. J. Gerontol. A Biol. Sci. Med. Sci..

[31] Ball H.C., Levari-Shariati S., Cooper L.N., Aliani M. (2018). Comparative metabolomics of aging in a long-lived bat: Insights into the physiology of extreme longevity. PLoS ONE.

[32] Brunet-Rossinni A.K. (2004). Reduced free-radical production and extreme longevity in the little brown bat (Myotis lucifugus) versus two non-flying mammals. Mech. Ageing Dev..

[33] Huang Z., Jebb D., Teeling E.C. (2016). Blood miRNomes and transcriptomes reveal novel longevity mechanisms in the long-lived bat, Myotis myotis. BMC Genom..

[34] Seim I., Fang X., Xiong Z., Lobanov A.V., Huang Z., Ma S., Feng Y., Turanov A.A., Zhu Y., Lenz T.L. (2013). Genome analysis reveals insights into physiology and longevity of the Brandt’s bat Myotis brandtii. Nat. Commun..

[35] Rasweiler J.J.t., Badwaik N.K. (1996). Improved procedures for maintaining and breeding the short-tailed fruit bat (*Carollia perspicillata*) in a laboratory setting. Lab. Anim..

[36] Rasweiler Iv J.J., Badwaik N.K., Barnard S.M. (2009). The laboratory environment for maintaining and breeding some bats in the Family Phyllostomidae. Bats in Captivity.

[37] Skrinyer A.J., Faure P.A., Dannemiller S., Ball H.C., Delaney K.H., Orman R., Stewart M., Cooper L.N. (2017). Care and husbandry of bats, the world’s only flying mammals. Lab. Anim. Sci. Pro..

[38] Rasweiler J.J.t., Badwaik N.K., Mechineni K.V. (2011). Ovulation, fertilization, and early embryonic development in the menstruating fruit bat, *Carollia perspicillata*. Anat. Rec..

[39] Rasweiler J.J.t., Cretekos C.J., Behringer R.R. (2009). The short-tailed fruit bat *Carollia perspicillata*: A model for studies in reproduction and development. Cold Spring Harb. Protoc..

[40] Smith J.B., Alloway K.D., Hof P.R., Orman R., Reser D.H., Watakabe A., Watson G.D.R. (2019). The relationship between the claustrum and endopiriform nucleus: A perspective towards consensus on cross-species homology. J. Comp. Neurol..

[41] Paxinos G., Franklin K.B.J. (2004). The Mouse Brain in Stereotaxic Coordinates.

[42] Paxinos G., Watson C. (2007). The Rat Brain in Stereotaxic Coordinates.

[43] Ding S.L., Van Hoesen G.W. (2015). Organization and Detailed Parcellation of Human Hippocampal Head and Body Regions Based on a Combined Analysis of Cyto- and Chemoarchitecture. J. Comp. Neurol..

[44] Orman R. (2015). Claustrum: A case for directional, excitatory, intrinsic connectivity in the rat. J. Physiol. Sci..

[45] Committee for the Update of the Guide for the Care and Use of Laboratory Animals (2011). Guide for the Care and Use of Laboratory Animals.

[46] Rasweiler J.J.t., Cretekos C.J., Behringer R.R. (2009). Feeding short-tailed fruit bats (*Carollia perspicillata*). Cold Spring Harb. Protoc..

[47] Jebb D., Huang Z., Pippel M., Hughes G.M., Lavrichenko K., Devanna P., Winkler S., Jermiin L.S., Skirmuntt E.C., Katzourakis A. (2020). Six reference-quality genomes reveal evolution of bat adaptations. Nature.

[48] Zeidman P., Maguire E.A. (2016). Anterior hippocampus: The anatomy of perception, imagination and episodic memory. Nat. Rev. Neurosci..

[49] Taube J.S. (1993). Electrophysiological properties of neurons in the rat subiculum in vitro. Exp. Brain Res..

[50] Witter M.P., Groenewegen H.J. (1990). The subiculum: Cytoarchitectonically a simple structure, but hodologically complex. Prog. Brain Res..

[51] Harris E., Witter M.P., Weinstein G., Stewart M. (2001). Intrinsic connectivity of the rat subiculum: I. Dendritic morphology and patterns of axonal arborization by pyramidal neurons. J. Comp. Neurol..

[52] Kunitake A., Kunitake T., Stewart M. (2004). Differential modulation by carbachol of four separate excitatory afferent systems to the rat subiculum in vitro. Hippocampus.

[53] Orman R., Von Gizycki H., Lytton W.W., Stewart M. (2008). Local axon collaterals of area CA1 support spread of epileptiform discharges within CA1, but propagation is unidirectional. Hippocampus.

[54] Naggar I., Stewart M., Orman R. (2020). High Frequency Oscillations in Rat Hippocampal Slices: Origin, Frequency Characteristics, and Spread. Front. Neurol..

[55] Ding S.L., Yao Z., Hirokawa K.E., Nguyen T.N., Graybuck L.T., Fong O., Bohn P., Ngo K., Smith K.A., Koch C. (2020). Distinct Transcriptomic Cell Types and Neural Circuits of the Subiculum and Prosubiculum along the Dorsal-Ventral Axis. Cell Rep..

[56] Bienkowski M.S., Sepehrband F., Kurniawan N.D., Stanis J., Korobkova L., Khanjani N., Clark K., Hintiryan H., Miller C.A., Dong H.W. (2021). Homologous laminar organization of the mouse and human subiculum. Sci. Rep..

